# Case Report: Compound heterozygous *ITGB2* variants causing leukocyte adhesion deficiency type I With preserved CD18 expression

**DOI:** 10.3389/fimmu.2026.1861396

**Published:** 2026-08-06

**Authors:** Marwa Abdelbari, Zeineb Ben Lamine, Raoudha Kebaili, Hajer Ben Belgacem, Jaballah Nesrine, Najla Mekki, Imen Ben-Mustapha, Najla Soyah, Jihene Bouguila, Amel Tej, Lamia Boughammoura

**Affiliations:** 1 Department of Pediatrics, Farhat Hached Hospital, University of Sousse, Sousse, Tunisia; 2 Faculty of Medicine of Sousse, University of Sousse, Sousse, Tunisia; 3 Laboratory of Immunogenetics LR23ES06, Faculty of Medicine of Sousse, University of Sousse, Sousse, Tunisia; 4 Regional Blood Transfusion Center, Sousse, Tunisia; 5 Laboratory of Immunology, Institute Pasteur de Tunis and Faculty of Medicine, University Tunis El Manar, Tunis, Tunisia

**Keywords:** CD18 expression, immunodeficiency, ITGB2 heterozygous mutation, leukocyte adhesion deficiency, pediatrics

## Abstract

**Introduction** Leukocyte adhesion deficiency (LAD) type I is a well-recognized inborn error of immunity that typically presents in early childhood. However, cases with normal surface expression of adhesion molecules are rare and suggest that these molecules may be functionally impaired despite their preserved expression.

**Observation:**

We report the case of a 3-year-and-8-month-old female with a family history of LAD type I due to absent adhesion molecule expression. The patient experienced recurrent hospitalizations since the neonatal period for multiple infections, predominantly ecthyma gangrenosum and purulent otitis media. Immunological analysis revealed normal surface expression of adhesion molecules, contrasting with her severe clinical and laboratory phenotype suggestive of LAD. Genetic testing identified two variants in *ITGB2*: a pathogenic deletion (c.119_128del) inherited from her healthy father and a missense variant (c.700G>A), classified as likely pathogenic, inherited from her healthy mother. Notably, the c.700G>A variant has not been previously reported in association with LAD syndrome. These findings suggest that the compoundsite heterozygosity of these two variants may be associated with the observed phenotype.

**Conclusion:**

LAD type I may present in children with apparently normal expression of adhesion molecules. Functional studies are warranted to assess the impact of the c.700G>A variant, which may affect protein function despite preserved expression.

## Introduction

Leukocyte adhesion and migration are essential processes for the immune response, enabling leukocytes to reach sites of infection and inflammation (1, 2). These processes rely on a tightly regulated interplay between selectins, chemokines, and integrins (1, 2). Selectins expressed on activated endothelial cells mediate the initial rolling of leukocytes along the vascular endothelium. Subsequent chemokine-induced inside-out signaling triggers conformational changes in LFA-1 (αLβ2 integrin), allowing it to transition from a bent, low-affinity state to an extended, high-affinity conformation capable of binding endothelial ICAM-1 and ICAM-2. Stable adhesion then initiates outside-in signaling, promoting cytoskeletal reorganization and transendothelial migration (1, 2).

Inherited defects in this adhesion cascade result in inborn errors of immunity known as leukocyte adhesion deficiencies (LAD) (3). LAD type I (LAD I), the most common and severe form, is caused by mutations in *ITGB2*, encoding the β2 integrin subunit (CD18). Clinically, it is characterized by recurrent life-threatening bacterial infections, delayed umbilical cord separation, skin ulcers, impaired wound healing, and marked leukocytosis (4, 5). LAD type II arises from defective fucosylation of selectin ligands, impairing leukocyte rolling, and is often associated with growth and neurodevelopmental delays with hh-blood group (4, 5). LAD type III, caused by mutations in *FERMT3*, disrupts inside-out integrin activation, leading to susceptibility to infections combined with platelet dysfunction and bleeding tendencies (4, 5).

Here, we describe a 3-year-old girl with classic clinical features of LAD I, including recurrent severe infections, persistent hyperleukocytosis, failure to thrive, and developmental delay, despite multiple evaluations showing normal CD18 surface expression. This unusual presentation delayed the diagnosis and highlights the need for careful clinical and genetic evaluation in suspected LAD I, even when conventional immunophenotyping is normal.

## Case presentation

**Figure 1:**
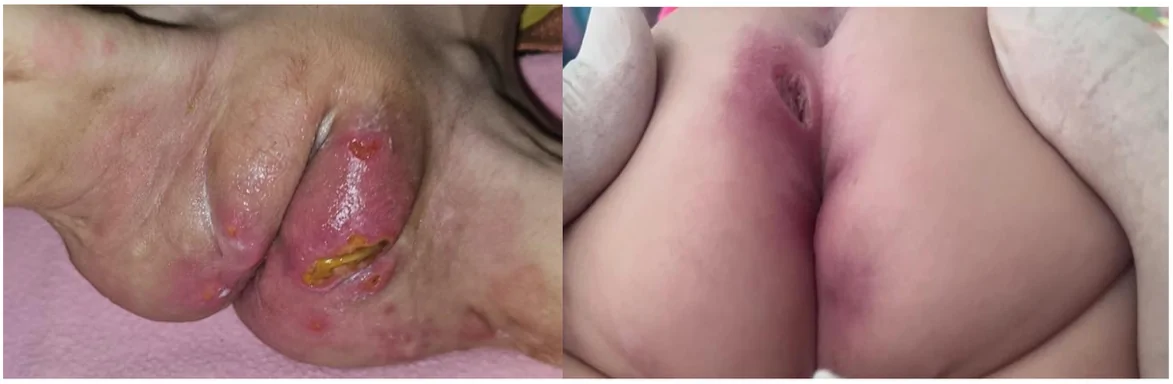
Ecthyma gangrenosum of the perineum caused by *Pseudomonas aeruginosa*.

We report the case of a 3-year-and-8-month-old female (P1) born to non- consanguineous parents, with a significant family history of LAD. Her sister (P2) died at 17 days of age from severe sepsis with marked hyperleukocytosis (138,000 WBC/mm³) and delayed umbilical cord separation. In her extended family, a maternal uncle with delayed cord separation died at 3 months, and a maternal cousin was previously diagnosed with LAD type I following neonatal meningitis, suppurative omphalitis, and pulmonary infections, confirmed by absent CD18 expression as assessed by flow cytometry (**Supplementary Figure**) This cousin underwent bone marrow transplantation (BMT) with a favorable clinical outcome. P1 was first hospitalized during the neonatal period for acute fever at 5 days of age, with a urinary tract infection due to *Candida albicans*. At 1½ months, she developed bacterial meningitis (pleocytosis 200 cells/mm³, 88% neutrophils), although no causative organism was identified. Since then, she has required multiple hospitalizations for severe and recurrent infections affecting various sites, including urinary tract infections and bacteremia caused by *Candida albicans, Klebsiella pneumoniae, Serratia marcescens, Enterococcus faecium, and Escherichia coli*, recurrent ecthyma gangrenosum with isolation of *Pseudomonas aeruginosa*, bilateral pyocyanic otitis media, digestive infections, perianal and labial abscesses, adenitis, and acute ethmoiditis. Recurrent skin infections (**Figure 1**), particularly ecthyma gangrenosum involving the buttocks, became a prominent feature of her clinical course. Severe growth retardation and periodontitis were also present. All episodes were accompanied by marked leukocytosis, reaching 100,000 WBC/mm³. This clinical and biological presentation, together with her family history, was unambiguously suggestive of a leukocyte adhesion defect.

**Figure 2:**
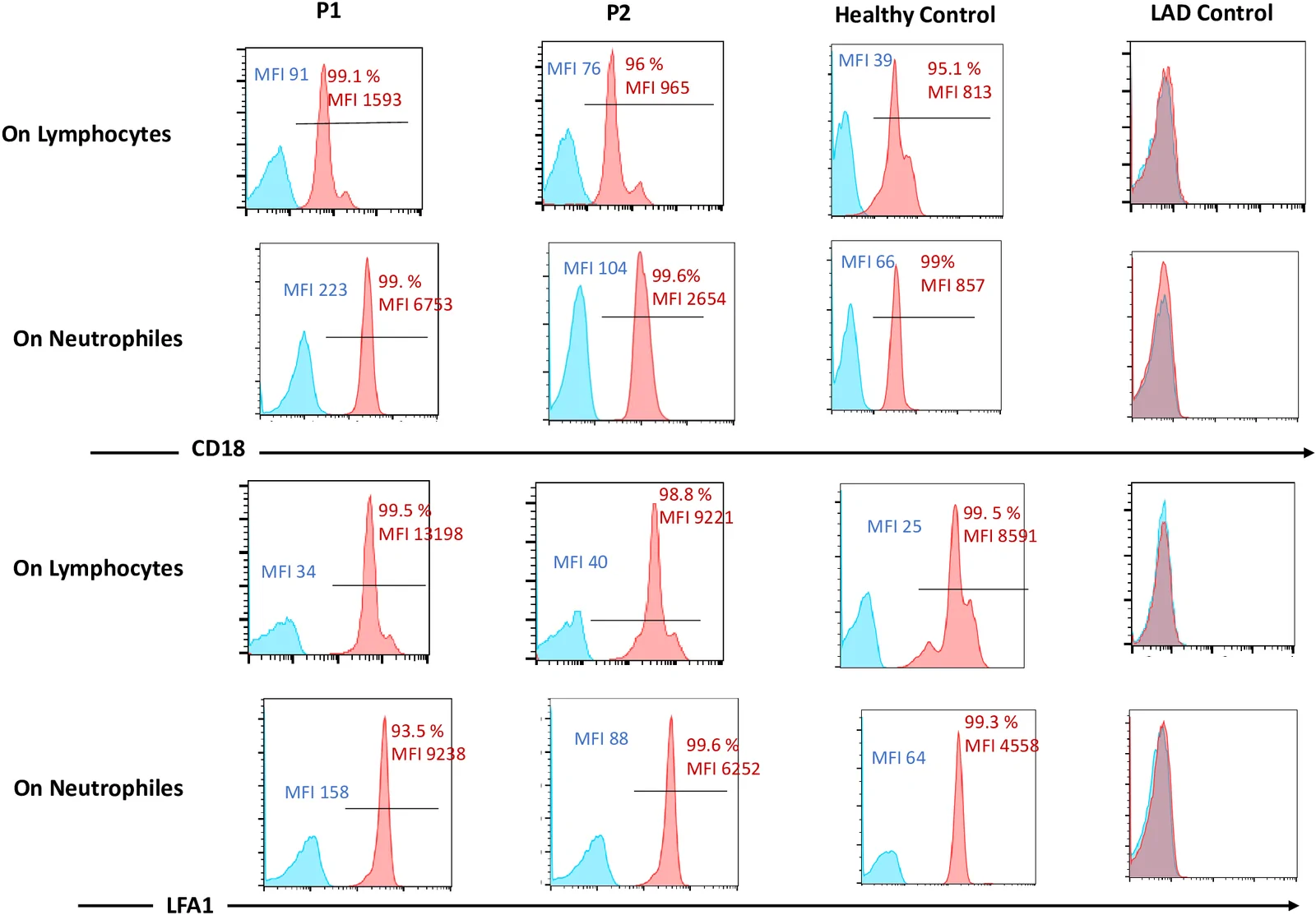
Flow cytometry analysis of CD11/CD18 expression on lymphocytes and neutrophils: “P1: index case; P2: sister; healthy control; LAD control: A female relative previously diagnosed leukocyte adhesion deficiency type I patient with absent CD18/CD11a expression, included as a negative control for β2-integrin expression.

Immunological evaluation revealed normal lymphocyte immunophenotyping with normal surface expression of adhesion molecules confirmed in two independent experiments (LFA-1: 99.5%, CD18: 99.5%) (**Table 1**, **Figure 2**). Serum immunoglobulin levels (IgG, IgA, IgM) and complement components were within age-adjusted reference ranges. The nitroblue tetrazolium (NBT) test was normal, indicating preserved neutrophil oxidative burst activity. The nitroblue tetrazolium (NBT) reduction test was performed using lipopolysaccharide (LPS) stimulation to assess neutrophil oxidative burst activity and exclude chronic granulomatous disease. Because of the strong clinical suspicion despite normal expression studies, whole genome sequencing was performed. This analysis identified two variants in the *ITGB2* gene (NM_000211.5), potentially in a composite heterozygous state: a heterozygous deletion (c.119_128del), and a missense variant (c.700G>A). The 10-nucleotide deletion leads to a frameshift and a premature stop codon at position 7 downstream. The predicted consequence is truncation of the β2 integrin protein. According to American College of Medical Genetics and Genomics (ACMG) guidelines, this variant is classified as pathogenic (PVS1, PM2, PP3). The missense variant results in the substitution of glutamic acid by lysine at codon 234 within the I-like domain of the β2 integrin. The variant is absent from population databases including gnomAD (v2.1.1 and v4.1.0), ClinVar, and the Leiden Open Variation Database V.3.0 (LOVD). The variant was also absent from an in-house database of 100 Tunisian individuals analyzed by whole-exome sequencing. In silico prediction tools consistently support a deleterious effect of the c.700G>A variant, including SIFT (0.00), PolyPhen-2 (0.996–1.000), REVEL (0.818), CADD Phred (25.1), and AlphaMissense (0.995); splicing effect was excluded by SpliceAI (all scores ≤ 0.04). (**Supplementary Table 1**). Familial segregation analysis by Sanger sequencing of the *ITGB2* gene confirmed compound heterozygosity, with paternal transmission of the pathogenic c.119_128del variant and maternal transmission of the c.700G>A missense variant (**Table 2**). Integrating these findings, the c.700G>A variant was classified as likely pathogenic according to ACMG/AMP guidelines (**Supplementary Table 2**). Additionally, a heterozygous variant in *ALG2* (c.1055_1056del, p.Ser352TyrfsTer2) was identified, classified as likely pathogenic according to ACMG criteria. The patient is a heterozygous carrier of this variant; it is important to note that the conditions associated with biallelic *ALG2* variants, namely congenital disorder of glycosylation type II and myasthenic syndrome, follow an autosomal recessive inheritance pattern, and heterozygosity alone is not expected to cause these phenotypes Analysis of genes associated with inborn errors of immunity did not identify any additional variants of potential clinical significance beyond those reported here. This patient is currently being treated with intravenous antibiotics for her documented infections. Since HLA typing did not reveal a genoidentical donor, the patient is being prepared for a haploidentical hematopoietic stem cell transplantation.

**Table 1:** Lymphocyte immunophenotyping results.

Parameter	Percentage (%)		Absolute Count (cells/µL)		Reference range (cells/µL)
	P1	P2	P1	P2	
Global WBC count	-	-	95240	102630	
CD3+ T lymphocytes	86.52	73.78	11 507	8133	[2500-5600]
CD3+ CD4+ T cells	51.05	53.76	7 106	5655	[1600-4000]
CD3+ CD8+ T cells	33.80	17.73	4705	1865	[560-1700]
CD19+ B lymphocytes	4.79	12.88	608	1485	[300-2000]
CD16+ CD56+ NK cells	8.04	12.03	1019	1387	[170-1100]
LFA-1 expression	99.5	99	**-**	**-**	**-**
CD18 expression	99.5	96	**-**	**-**	**-**

**Table 2:** Genetic analysis of leukocyte adhesion deficiency mutations.

Family members	Gene	Genomic position	Variant	Amino acid	Classification	Status
Index child	*ITGB2* *ALG2*	Chr21:46330218 Chr9:101980411	c.119_128del c.700G>A c.1055_1056del	p.Gly40AlafsTer7 p.Glu234Lys p.Ser352TyrfsTer2	Pathogen Likely pathogenic Likely pathogenic	Detected Detected Heterozygous
father	*ITGB2*	Chr21:46330218	c.119_128del	p.Gly40AlafsTer7	Pathogenic	Heterozygous
mother	*ITGB2*	Chr21:46330218	c.700G>A	p.Glu234Lys	Likely pathogenic	Heterozygous

## Discussion

LAD I is a rare but severe inborn error of immunity characterized by recurrent life-threatening bacterial and fungal infections, impaired wound healing, delayed umbilical cord separation, marked leukocytosis, and absence of pus formation (5). More than 150 pathogenic variants have been identified in *ITGB2* (OMIM *600065), including deletions, insertions, nonsense, frameshift, and missense mutations (6, 7). The *ITGB2* gene encodes the β2 integrin subunit, which associates with α subunits (CD11a, CD11b, CD11c, and CD11d) to form the heterodimeric integrins LFA-1 (αLβ2), Mac-1 (αMβ2), p150,95 (αXβ2), and αDβ2. These integrins are essential for leukocyte rolling, firm adhesion, and transendothelial migration through interactions with endothelial ligands such as ICAM-1 to ICAM-5 and JAM-A (2). Most pathogenic mutations cluster within a highly conserved ~240-amino-acid region encoded by exons 5–9, corresponding to the βI (I-like) domain, which contains the metal ion-dependent adhesion site (MIDAS) and plays a critical role in ligand binding and integrin activation (6). In most cases, *ITGB2* mutations lead to absent or markedly reduced expression of β2 integrins; Disease severity correlates with residual CD18 surface expression and is commonly classified as severe (<2%), moderate (2–30%), or mild (>30%) (3). However, a subset of patients exhibits normal CD18 expression with profound functional impairment, representing a diagnostic challenge (6, 7). In a review of 139 reported LAD I cases, 18 patients exhibited CD18 expression levels above 30% (range: 31–99%). Among the 12 cases for which corresponding CD11 data were available, 8 showed expression of at least one CD11 subunit below 2% (7). Our patient exemplifies this atypical presentation: repeated immunophenotyping showed normal CD18 and CD11a expression, which initially led to the exclusion of LAD I despite a highly suggestive clinical phenotype. This emphasizes that clinical suspicion must guide further genetic evaluation, even when conventional assays appear normal.

Genetic analysis revealed a compound heterozygous genotype: a frameshift deletion c.119_128del (p.Gly40AlafsTer7) inherited from the father, and a novel missense variant c.700G>A (p.Glu234Lys) inherited from the mother. The c.119_128del variant results in a frameshift with a premature termination codon, predicting a complete loss of CD18 function. This variant was classified as pathogenic according to ACMG criteria (8–10). In silico prediction tools support a deleterious effect (CADD >30, MutationTaster: disease causing) (11, 12). Importantly, this deletion has been previously reported in homozygous form as causative of severe LAD I and has been associated with complete absence of CD18 expression (13). As highlighted by Smith et al., among three patients carrying this deletion, only one exhibited CD18 expression of approximately 50%, while showing an absence of CD11a. Smith et al. therefore suggest that the inclusion of CD11a analysis, together with CD18, may improve the detection of LAD-I and help prevent delayed diagnosis, given that CD11a was nearly absent in all patients in their cohort (13). In our study, CD11a expression was preserved in both patients (P1 and P2) despite the presence of the *ITGB2* deletion, suggesting a more complex relationship between genotype and surface integrin expression. The elevated absolute T-cell counts observed in both affected siblings may reflect altered lymphocyte trafficking secondary to impaired β2-integrin function. Although leukocytosis in LAD is predominantly attributed to defective neutrophil extravasation, LFA-1 also plays a central role in T-cell adhesion, transendothelial migration, and tissue homing (14). While the functional consequences of homozygous *ITGB2* deletions on CD18 expression have been well established, the specific impact of the c.119_128del deletion particularly in the heterozygous state or in potential compound heterozygosity has not yet been fully elucidated.

The second variant, p.Glu234Lys, is a missense substitution resulting in the replacement of glutamic acid (E), a highly conserved residue in vertebrates, by lysine (K) at position 234 within the βI-like domain of the β2 integrin chain, a region critical for integrin activation. This variant is absent from population databases (PM2) ClinVar, and the LOVD database (8) and was not detected in a whole-exome sequencing cohort of 100 Tunisian patients evaluated for unrelated genetic conditions. In silico predictions are suggesting deleterious effects. To our knowledge, this c.700G>A (p.Glu234Lys) variant has not been previously reported in association with LAD syndrome, representing the first description of this missense change in the context of a leukocyte adhesion deficiency phenotype. Based on current evidence, including rarity in population databases and in silico predictions, this variant has been classified as a likely pathogenic according to ACMG criteria (10). Notably, this residue has been suggested to participate in Ca²^+^ coordination at the LIMBS (Ligand-Induced Metal-Binding Site) and Mg²^+^ coordination at the MIDAS site, implicating a potential role in metal-dependent integrin conformational regulation (https://www.uniprot.org). Mathew et al. described a compound heterozygous LAD I patient harboring a p.Asp231His mutation, located in close proximity to p.Glu234, who exhibited normal surface expression of LFA-1, Mac-1, and p150,95, but a complete loss of adhesion to physiological ligands (15). Given the structural proximity of p.Asp231 to p.Glu234, it is possible that the p.Glu234Lys variant similarly disrupts integrin activation, leading to a functional defect of β2 integrins in our patient, despite potentially preserved surface expression (15). However, we acknowledge that functional validation including cellular adhesion assays and transmigration remains warranted to confirm the loss-of-function effect on CD18, and this is recommended as a priority for future studies.

Additionally, the patient carried a heterozygous frameshift variant in *ALG2* (c.1055_1056del, p.Ser352TyrfsTer2), classified as likely pathogenic according to ACMG criteria (16). While heterozygous *ALG2* variants alone do not cause congenital disorders of glycosylation, defects in glycosylation pathways are known contributors to LAD syndromes, such as mutations in *SLC35C1* (3). Given that β2 integrins are highly glycosylated, defects in glycoprotein processing may compromise their maturation or function, and the presence of multiple immune-relevant variants may further influence the clinical severity of LAD.

Hematopoietic stem cell transplantation (HSCT) remains the only curative treatment for severe LAD I and LAD III. However, HSCT is limited by donor availability and the risk of graft-versus-host disease. In our patient, HLA typing did not identify an HLA-genoidentical donor; therefore, HSCT is planned in the near future using a haploidentical donor, namely the patient’s father. Recent advances in gene therapy and genome editing offer promising alternatives. Notably, CRISPR-Cas9–mediated correction of a 10-bp deletion in exon 3 of *ITGB2* has recently been shown to restore CD18 expression in patient-derived cells, highlighting the potential of targeted gene correction as a future therapeutic strategy for LAD I (17).

## Conclusion

In conclusion, this case illustrates a severe form of LAD I caused probably by a compound heterozygous *ITGB2* genotype combining a frameshift mutation and a missense variant affecting a critical functional domain. The findings emphasize that LAD I can result from both quantitative and qualitative defects in CD18. This case highlights that normal CD18 expression does not exclude LAD I and underscores the need for genetic evaluation in highly suggestive clinical contexts. Functional studies are warranted to confirm the impact of the c.700G>A variant, which may abrogate molecular function despite preserved expression.

## Data Availability

The datasets presented in this study can be found in online repositories. The names of the repository/repositories and accession number(s) can be found below: https://www.ncbi.nlm.nih.gov/, SCV007539359.
