# Supplementary material for: Case Report: Compound heterozygous ITGB2 variants causing leukocyte adhesion deficiency type I With preserved CD18 expression
Source: Front Immunol. 2026 Aug 6;17:1861396. doi: 10.3389/fimmu.2026.1861396 (PMC13489901; doi:10.3389/fimmu.2026.1861396)
Supplement: Supplementary file 1 [file Table1.pdf]

Supplementary Table 1 – In silico predictions for ITGB2 variant (700G>A)

TOOL / DATABASE	SCORE / RESULT	THRESHOLD	PREDICTION
**POPULATION DATABASE**			
gnomAD exome<br>v2.1.1<br>v4.1.0	*Not observed*	—	Absent
gnomAD genome<br>v2.1.1<br>v4.1.0	*Not observed*	—	Absent
ClinVar germline	*No match*	—	Absent
LOVD	*No match*	—	Absent
In-house Tunisian WES	*Not observed*	—	Absent
**CONSERVATION**			
phyloP (239 primates)	0.663	≥ 0.304	Conserved
phyloP (241 vertebrates, cactus)	2.783	≥ 1.345	Conserved
GPN-MSA	−9.33	≤ −7	Deleterious
**PROTEIN IMPACT — INDIVIDUAL PREDICTORS**			
SIFT	0.00	< 0.05	Deleterious
PolyPhen-2 HumDiv	1.000	≥ 0.957	Prob. damaging
PolyPhen-2 HumVar	0.996	≥ 0.909	Prob. damaging
Fathmm-XF	0.921	≥ 0.5	Damaging
AlphaMissense	0.995	≥ 0.564	Likely pathogenic
CADD (Phred)	25.10	≥ 25	Damaging
**PROTEIN IMPACT — META-PREDICTORS**			
REVEL	0.818	≥ 0.75	Damaging
ClinPred	0.990	≥ 0.5	Damaging
MetaSVM	0.966 (RI: 9)	≥ 0	Damaging
MetaLR	0.921 (RI: 9)	≥ 0.5	Damaging
Mistic	0.97	≥ 0.5	Damaging

TOOL / DATABASE	SCORE / RESULT	THRESHOLD	PREDICTION
**POPULATION DATABASE**			
BayesDel (with MAF)	0.273	≥ 0.13	Likely pathogenic
MPA	8.0 / 10	High ≥ 7	High missense impact
**SPLICING PREDICTIONS**			
SpliceAI (AG / AL / DG / DL)	0.00 / 0.00 / 0.04 / 0.00	≥ 0.2 (low), ≥ 0.5 (med), ≥ 0.8 (high)	No splice impact
SPiP	5.03% [2.44–9.05%]	< 10% = low risk	No splice impact
AbSplice	< 0.01	≥ 0.01	No splice impact
MaxEntScan (5'ss / 3'ss)	No significant score	—	No splice impact
dbscSNV (ADA / RF)	*No match*	≥ 0.8	No splice impact

Abbreviations: AG, acceptor gain; AL, acceptor loss; DG, donor gain; DL, donor loss; MAF, minor allele frequency; MPA, Missense variant Prioritization tool with Aggregated score; RI, reliability index; SPiP, Splicing Prediction in Pathology; WES, whole-exome sequencing.
All scores retrieved from MobiDetails (accessed 24 May 2026). Variant notation: NM_000211.5:c.700G>A, p.(Glu234Lys); hg38: chr21:g.44,901,533C>T; hg19: chr21:g.46,321,448C>T. Position within exon 6, 42 bp from the donor splice site; domain: VWFA (residues 124–363, UniProt P05107).

Supplementary Table 2. ACMG/AMP variant classification of the *ITGB2* missense variant (700G>A)

CRITERIA	EVIDENCE	STRENGTH
PM1	Missense variant located within the βI (I-like) domain of CD18 (integrin β2), a critical functional domain involved in ligand binding	Moderate
PM2	Absent in population databases	Supporting
PM3	Detected *in trans* with a validated pathogenic *ITGB2* variant	Moderate
PP3	In silico predictions support a deleterious effect	Supporting

PP4	Patient phenotype is highly specific and consistent with Leukocyte Adhesion Deficiency type I	Supporting
Final ACMG/AMP classification		Likely Pathogenic
